# Complete Genome Sequence of “*Candidatus* Viadribacter manganicus” Isolated from a German Floodplain Area

**DOI:** 10.1128/genomeA.00897-16

**Published:** 2016-09-01

**Authors:** Burga Braun, Ulrich Szewzyk

**Affiliations:** Technische Universität Berlin, Chair of Environmental Microbiology, Berlin, Germany

## Abstract

Iron- and manganese-depositing bacteria occur in many soils and all water systems, and their biogenic depositions of ochre in technical systems may cause severe clogging problems and monetary losses. “*Candidatus* Viadribacter manganicus” is a small coccoid, iron- and manganese-depositing bacterium isolated from the Lower Oder Valley National Park, Germany.

## GENOME ANNOUNCEMENT

The suspended biofilm was spread on ATA medium based on Mn-Agar (1): 2 g·L^−1^ MnCO_3_ hydrate, 0.15 g·mL^−1^ trisodium citrate, 0.2 g·L^−1^ Fe(NH_4_)_2_(SO_4_)_2_, 0.1 g·L^−1^ cycloheximide, 2-mL vitamin solution (2), 0.338 g·L^−1^ H_5_CN × HCl, 2 mL trace element solution SL 9 (3), 20 g agar, and 1 liter filtered water from Lake Daminke, the source of the biofilm. After an incubation time of up to 2 weeks at room temperature, we selected dark brown colonies that formed on the agar surface. Iron- and manganese-deposition of the strain was confirmed using the methods of Schmidt et al. (4). EDX mapping of colonies suggested that manganese and iron were predominantly concentrated outside the bacteria cells. Determination of the physiological characteristics using BIOLOG GN2 micro plates (I&L Biosystems GmbH, Königswinter, Germany) showed that strain A272 was only able to metabolize the N-actelyl-D-galactoseamine and propionic acid. The strain was deposited in the publicly accessible culture collections DSMZ and LMG under the accession numbers DSM 25961, and LMG 27107.

Total genomic DNA was extracted using the GeneMATRIX soil DNA purification kit (Roboklon, Berlin, Germany). The paired-end library was prepared by using a TruSeq DNA HT sample prep kit (Illumina Nehterlands, Eindhoven) and mate-pair libraries were established using the Nextera mate-pair sample preparation kit (Illumina Netherlands, Eindhoven). Genome sequencing was done on an Illumina MiSeq sequencer by generating 7,167,266 paired-end and 4,324,110 mate-end reads. For read trimming, base correction, and *de novo* assembly, SPADES 3.5; A5 20140604, CLC Genomic workbench, and Geneious 8 software were utilized. The draft genome included five contigs with one scaffold containing two gaps, that were filled by Sanger sequencing, producing the complete genome. The shortest sequence was 5,392 bp and the longest sequence was 1,826,082 bp. The total size of the scaffold/draft genome was 3,733,622 bp and the complete genome was 3,732,719 bp with a G+C content of 61.5%. The genome was annotated using the NCBI Prokaryotic Genome Annotation Pipeline version 3.0 resulting in 3,848 genes and 48 pseudogenes, seven tandem clustered regularly interspaced short palindromic repeat (CRISPR) repeat units, three rRNAs (5S 0.16 S, 23 S) and three complete rRNAs (5S 0.16 S, 23 S), 53 tRNAs, and one noncoding RNA (ncRNA). BLASTn searches (5) excluding models and uncultured sample sequences revealed a 91% 16S rRNA gene sequence similarity to *Hyphomonadaceae bacterium* UKL13-1 (CP012156.1) and 89% similarity to *Maricaulis maris* MCS 10 (CP000449.1), *Hyphomonas neptunium* ATCC 15444 (CP000158.1), *Rhodobacter sphaeroides* ATCC 17025 (CP000663.1), and *Brevundimonas naejangsanensis* strain B1 (CP015614.1).

### Accession number(s).

This whole-genome shotgun project has been deposited in GenBank under the accession number CP013244. The version described in this paper is the first version.
